# Relationship between socio-descriptive characteristics, burnout syndrome, and quality of life of employees

**DOI:** 10.3389/fpubh.2023.1277622

**Published:** 2024-03-07

**Authors:** Natasa K. Rancic, Dejan R. Veljkovic, Momcilo R. Mirkovic, Ljiljana M. Kulic, Verica S. Jovanovic, Bojana N. Stamenkovic, Natasa S. Maksimovic, Vojislav M. Ciric, Emilija M. Marinkov-Zivkovic, Sonja D. Giljaca, Gordana Đorđevic, Ognjen G. Đorđevic, Marko M. Stojanovic, Novica Z. Bojanic, Dusan P. Miljkovic, Suzana A. Otasevic

**Affiliations:** ^1^Faculty of Medicine Nis, University of Nis, Nis, Serbia; ^2^Institute for Public Health Nis, Nis, Serbia; ^3^Ministry of Internal Affairs, Gendarmerie Detachment in Kraljevo, Kraljevo, Serbia; ^4^Faculty of Medicine, University of Pristina, Kosovska Mitrovica, Serbia; ^5^Institute of Public Health of the Republic of Serbia, Belgrade, Serbia; ^6^Institute Niska Banja, University Clinical Centre Nis, Nis, Serbia; ^7^Faculty of Medicine Belgrade, University of Belgrade, Institute of Epidemiology, Belgrade, Serbia; ^8^University Clinical Centre Nis, Nis, Serbia; ^9^City Pubic Institute of Public Health Belgrade, Belgrade, Serbia; ^10^Institute of Public Health Kragujevac, Kragujevac, Serbia; ^11^Faculty of Medical Sciences, University of Kragujevac, Kragujevac, Serbia

**Keywords:** burnout syndrome, security employees, professional private sector, quality of life, Central Serbia

## Abstract

**Introduction:**

Burnout syndrome develops as a consequence of chronic stress among employees. The study objective was to examine what socio-descriptive characteristics of employees might be associated with the appearance of the occupational burnout and to evaluate the relationship between job burnout and the quality of life among security employees of the professional private security sector in Central Serbia.

**Methods:**

A multicenter cross-sectional questionnaire-based study was performed. A multivariate logistic regression analysis and ANOVA post choc test was applied.

**Results:**

A total of 353 respondents (330 male and 23 female) participated in the study. Female sex and older age were associated with a higher risk of total burnout and the development of emotional exhaustion while male sex, higher education, and managerial position were associated with higher personal achievement and lower risk of total burnout. Male sex, marital union, two or more children, and direct contact with clients were significantly associated with a lower quality of life of employees. A significant negative correlation was found between total burnout and the Physical Health Composite Score (PHC) score with a correlation coefficient (*r*_s_) of −0.265 (95%CI from −0.361 to −0.163); between total burnout and the and Mental Health Composite Score (MHC) score with a *r*_s_ of −0.391 (95%CI from −0.480 to −0.301); and between total burnout and TQL score with a *r*_s_ of −0.351 (95%CI from −0.445 to −0.258).

**Conclusion:**

Female sex and older age were associated with a higher risk of total burnout and the development of EE while a managerial position and higher education were protective factors in relation to the development of burnout. Male sex, marital union, two or more children, and direct contact with clients were significantly associated with a lower quality of life of the employees. Shift work significantly reduced the total quality of life, while managerial positions increased the quality of life.

## 1 Introduction

The police job is considered stressful, leading to poor physical and mental health outcomes over time, in which burnout is included (1, 2). Working in the private security sector is most similar to police work in most countries, whereby security employees have almost the same responsibilities and perform similar tasks (3, 4). Russell et al. (5) stated that security work is the most stressful job and because of that security officers suffer more burnout and illnesses than other employees do (5). Private security is as stressful as public security (6). Burnout syndrome develops as a consequence of chronic stress among employees, particularly those who perform high-risk jobs or care and protection jobs or jobs involving helping other people (7). Christiana Maslach defined burnout as “a psychological syndrome of emotional exhaustion (EE), depersonalization (DP), and a lowered sense of personal accomplishment (PA) that can be observed in people working with others in a certain specific manner” (8).

Emotional exhaustion is characterized by a sense of emotional overextension as a result of work; depersonalization is characterized by emotional indifference and dehumanization of service recipients; diminished sense of personal accomplishment is characterized by a sense of professional stagnation, incapability, incompetence, and unfulfillment. DP refers to the development of an insensitive and cynical attitude toward people who are service recipients, a negative attitude toward work, and the loss of a sense of own identity. A diminished sense of PA refers to a negative self-assessment of competences and accomplishments at a workplace, and the symptoms manifest as a lack of motivation to work, as well as a decline in self-esteem and general productivity (9, 10).

In the United States of America (USA), the prevalence of burnout was explored in a sample of 13,000 police officers from across 89 police agencies, describing that 19% of the sample felt emotionally exhausted on a weekly basis, and 13% had severe levels of depersonalization (11) describing that 19% of the sample felt emotionally exhausted on a weekly basis and 13% had severe levels of depersonalization. In Sweden (12), in a sample of 856 patrolling police officers, 28% reported high levels of emotional exhaustion and 56% increased levels of depersonalization. In Spain, in a sample of 747 and in police officers and guards in prisons in Spain, the percentage of those with burnout was 43.6% (13). According to the results of Lastvikova et al. (13), in the European Union (EU), about 8% of the German working population believe they suffer from burnout syndrome (14). In Costa Rica, the prevalence of total burnout was 44.4% (15).

According to Aguayo et al. (16), the role of socio-demographic factors in the development of the burnout syndrome is important because of: these factors might have a direct influence on burnout levels and its subscales and second, the identification of risk factors may define a risk profile of an employees and would facilitate the development of primary and secondary intervention programs.

Prior studies about burnout generally showed a male–female difference (17) although the results are inconsistent. Some studies have shown that women suffer more from burnout than men (18, 19), while others have indicated that males report higher burnout levels (20–23) and pointed out the influence of underlying, job-related factors such as working time hours (21). De Solana reported that being younger contributed to increased burnout levels (18) and the results of some studies point to an inverse relationship between age and burnout, such that people will experience lower levels of burnout as their age increases (12, 24).

A systematic review of the determinants of job burnout (25) found a significant relationship between increasing age and increased risk of DP; on the other hand, there was also a higher level of PA. Some researchers have found the opposite, namely that older employees are more at risk (26, 27). It is well known that single individuals are more at risk than those who live with a partner (28, 29) or are married. The number of children in a family can also play an important role in burnout (30–33). According to the interesting findings of Dutch researchers (32), the presence of young children and doing more household chores were positively related to feelings of burnout, whereas having children reduced employees’ feelings of burnout. In a study by Brady et al. (33), a higher number of children was correlated with a lower level of burnout.

The majority of studies show that individuals with a higher level of education are more at risk (30, 34) and results are inconsistent too. A review investigating the risk factors for stress among police officers reported that officers who worked in big cities were more prone to higher levels of stress and post-traumatic stress disorder as they were more frequently exposed to violent and extreme situations (35).

Burnout has serious professional and personal consequences, including lack of professionalism (often sick-leaves, reduced efficiency at work, lack of interest, and non-collegiality), problems in communication with close persons, divorces, losing friends, alienation, and aggressiveness (33, 35), which has a negative impact on the employees. Several studies have concluded that employees with higher levels of burnout are more likely to suffer from a variety of physical health problems such as musculoskeletal pain, gastric alterations, cardiovascular disorders, headaches, insomnia, and chronic fatigue, as well as increased vulnerability to infections (36, 37) and job burnout reduce the quality of life among security employees.

The following hypothesis were tested:

*Hypothesis 1*: Males has significant higher level of total burnout and higher EE and PA subscale, and decreased quality of life than females.

*Hypothesis 2*: Older age is in the significant relationship with the higher burnout.

*Hypothesis 3*: Security employees who are married show higher levels of quality of life.

*Hypothesis 4*: Security employees without children or with one child had a significantly higher MHC score and total quality of life, compared to employees with two, three, or more children.

*Hypothesis 5*: Managerial position and higher education are in significant positive relation with higher PA subscale and with the increased quality of life.

*Hypothesis 6*: Shift working is in the significant negative relationship with the decreased quality of life.

*Hypothesis 7*: Job burnout has significant impact on the reduction on the quality of life of the security employees.

## 2 Materials and methods

### 2.1 Private security sector in Central Serbia

The main characteristics of the professional private security sector in Central Serbia are as follows: the third largest group of people under arms, with about 40,000 employees (38); employees have different levels of training and different levels of education; employees have low social and economic status. Employees in the professional private security sector are exposed to various risk factors that can cause stress for an individual. In a country such as Serbia, which has undergone economic, political, and social reforms, as well as military and police reforms, a somewhat different hierarchy is expected with regard to job stressors, compared to developed countries. It is impossible to remove all the workplace stressors within a private company, but it is necessary to identify the stressors in order to reduce exposure and prevent the occurrence and development of burnout syndrome (14, 39, 40).

### 2.2 Study design

A multicenter cross-sectional study was done. The study was conducted on a representative sample, the size of which was determined using http://www.psychologie.hhu.de/arbeitsgruppen/allgemeine-psychologie-und-arbeitspsychologie/gpower.html (41).

The study included security staff employed at private agencies in seven cities in Central Serbia: Nis, Kraljevo, Kragujevac, Cacak, Krusevac, Belgrade, and Novi Sad. The representative sample size was 439, the study strength was 80%, and the type 1 error probability α was 0.05. The study included 353 questionnaires that were completely filled out. The response rate was 80%. The study was performed in the period from March 3 to April 30, 2019.

The Ethics Committee of the Faculty of Medicine of the University of Pristina with temporary seat in Kosovska Mitrovica by the Decision No -09-972-1 dated September 10, 2018.

The study inclusion criteria were as follows: adults 18–65 years of age, citizens of the Republic of Serbia, and full-time employees, who were licensed to work in private security, had-finished the basic training for using weapons, and were working longer than 12 months. Exclusion criteria were as: staff in the process of obtaining the license, discontinuity at work longer than 1 year, long-term sick leaves or multiple workplace changes in the past 5 years, respondents recently exposed to major psychophysical trauma regardless of the private environment (illness or death of a close person, divorce…etc.), and refusal to participate in the study.

The total score of the each respondent was obtained by summing up the matrix with a specific key for each of the three previously mentioned subscales, and the total degree of occupational burnout is represented by a comprehensive scale calculated based on a precise formula. High level of burnout at work is reflected in high scores on the emotional exhaustion and depersonalization subscales and low scores on the personal accomplishment subscale.

This means that high scores on the EE and DP scales contribute to the burnout syndrome, while high scores on the professional accomplishment scale diminish it. A medium level of burnout is a reflection of mean scores on all three subscales. A low level of burnout at work is reflected in low scores on the EE and DP subscales and high scores on the PA subscale. The PA scale is relevant only if confirmed with the EE or DP scale.

#### 2.2.1 The Maslach burnout inventory human services questionnaire

This is an internationally accepted burnout measuring standard that measures three burnout subscales, and it is often used as a model for the evaluation of validity of other burnout risk assessment scales. We have used the questionnaire for staff employed at institutions who are in direct contact with people (Human Services Survey, MBI-HSS) with 22 variables (42).

In the Republic of Serbia, licenses for the Maslach Burnout Inventory Human Services Survey (MBI-HSS) questionnaire and the evaluation key, as well as usage permission, were obtained directly from the current license owners-the SINAPSA EDITION Company (license No. 2/2018, dated May 9, 2018).

The managers of private security agencies provided written approval for the research. All respondents were informed in detail about the research, and they signed the consent forms to participate in the study.

Maslach Burnout Inventory Human Services Survey consists of 22 questions that are subsequently used in the calculation of three subscales measuring different occupational burnout aspects.

It is a self-administered questionnaire. Each question consists of a series of statements expressing the degree of agreement with the expressed statements. The seven-point Likert scale was used in the study and responders assessed each item on this scale from 0 to 6 (0—never, 1—once a year or less, 2—once a month or less, 3—a few times a month, 4—once a week, 5—a few times a week, and 6—every day).

The total score of each respondent was obtained by summing the matrix with a specific key for each of the three previously mentioned subscales, and the total degree of occupational burnout was represented by a comprehensive scale calculated using a precise formula. A high level of burnout at work is reflected in high scores on the emotional exhaustion and depersonalization subscales and low scores on the personal accomplishment subscale. This means that high scores on the EE and DP scales contribute to burnout syndrome, while high scores on the professional accomplishment scale diminish it. A medium level of burnout is a reflection of mean scores on all three subscales. A low level of burnout at work is reflected in low scores on the EE and DP subscales and high scores on the PA subscale. The PA scale is relevant only if confirmed with the EE or DP scale.

#### 2.2.2 Short form 36 health survey

Short Form 36 Health Survey is the most commonly used general questionnaire that measures subjective health, i.e., the health-related quality of life (HRQoL). The questionnaire is a generic instrument for measuring health status and quality of life. It is standardized and very sensitive for assessing the overall impact of health on the quality of life. It consists of 36 questions covering a period of 4 weeks. It is designed to analyze self-ranking individual perception of health, functional status, and sense of well-being (43). The questions cover eight domains in the field of health: physical functioning, limitations due to physical health, body pain, general health, vitality, social functioning, limitations due to emotional problems, and mental health. When the total of each individual domain is calculated, the next step is calculating the Physical Health Composite Score (PHC) and Mental Health Composite Score (MHC), which are related to the quality of life.

The eight domains of this questionnaire were used to assess the category (dimension) of functional health and the sense of well-being; four for physical and four for mental status. Very important for measuring the physical health are the following dimensions: physical functioning, physical role, body pain, and general health. All individual scores and both composite scores can have values ranging from 0 to 100, where 0 is very poor quality of life and 100 is ideal quality of life resulting from good physical and mental health. Composite scores of physical and mental health are used to calculate the total quality of life (TQL) score. The scales of vitality and general health have good validity; and the questionnaire is used to compare different conditions and diseases, and it is considered the “gold standard” in assessing the quality of life related to health.

### 2.3 Statistical analyses

All analyses were conducted in the SPSS-version 22 program package. We calculated means, odds ratios (ORs), and confidence intervals (CIs), and we performed variance analysis (ANOVA). Repeated-measures ANOVA was applied to test the statistical significance of changes in values of the indicators of quality of life. For the comparison of numerical characteristics, we used Student’s *t*-test. The chi-squared test was used to compare frequencies between groups, or the Fisher test was used when the expected frequency was <5 in one of the cells. A univariate logistic regression analysis, followed by a multivariate one was used to assess the relationship between burnout and the socio descriptive characteristics of the respondents obtained from the questionnaires. Tukey’s test was used to analyze values between two samples. Spearman correlation analysis was used to assess the association of burnout and the quality of life of responders, and Spearman’s coefficient of rank correlation was calculated (*r*), too. A value of *p* < 0.05 was considered statistically significant.

## 3 Results

### 3.1 Socio-descriptive characteristics of the respondents

Of the total number of people contacted (439), a total of 353 respondents (330 male and 23 female) participated in the study. The response rate was 80%. Men represented 93.5% of all respondents, and they were significantly older than women: 44.09 ± 11.44 vs. 36.91 ± 7.92 years (*F* = 8.752; *p* = 0.003).

Overall, 224 (64.3%) respondents were married, about 1/3rd did not have children (33.7%), and more than half had completed high school (54.7%). A minority of the employees had a university degree (7.6%). There were 300 (78.00%) employees in service; about 22.7% held managerial positions, 288 (81.6%) worked in shifts, and most respondents (as many as 277; 78.5%), worked 8–12 h a day.

### 3.2 Burnout in security employees in the private sector

**Figure 1:**
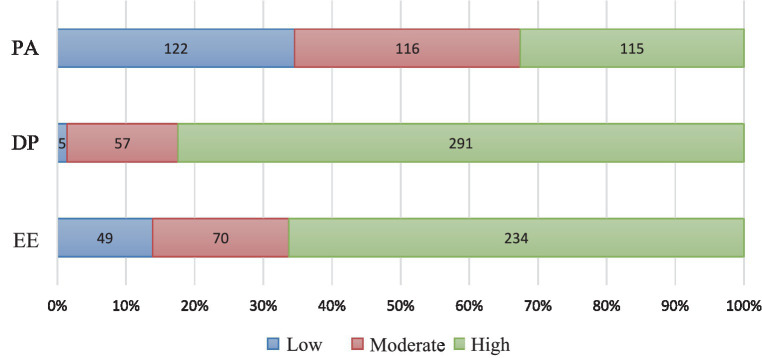
MBI-HSS subscales with regard to the severity of burnout symptoms. Reprinted with permission from Veljkovic et al. (44). © Dejan Veljkovic.

Figure 1 shows the values of subscales of burnout syndrome by severity—high, moderate, and low.

More than half of the employees *n* = 234, (66.3%) had a high level of EE, 19.8% (*n* = 70) had a moderate level and 13.9% (*n* = 49) had a low level of EE. Cumulatively, high and moderate levels of EE were present in 86.1% of employees. The largest number of employees, 82.4% (*n* = 291), had a high level of DP. Moderate level of DP was present in 16.2% (*n* = 57) and low level in 1.4% (*n* = 5) of employees. Low PA was recorded in 34.5% (*n* = 122), moderate in 32.9% (*n* = 116), and high in 32.6% (*n* = 115) of employees (Figure 1).

For each subscale of burnout (EE, DP, and PA) and for total burnout, multivariate logistic regression analysis was performed, and the results are presented in Table 1.

**Table 1:** Results of multivariate logistic regression analyses of dependent variables ЕЕ, DP, and PA, as well as total burnout.

		Emotional exhaustion (ЕЕ)		Depersonalization (DP)		Personal accomplishment (PA)		Total Burnout	
Characteristics		OR	95%CI	OR	95%CI	OR	95%CI	OR	95%CI
Sex	Female (reference value)								
	Male	0.277	0.081–0.951	0.689	0.198–2.395	2.644	0.879–7.954	0.303	0.088–1.041
Age		0.962	0.943–0.982	0.989	0.898–1.005	0.997	0.955–1.048	1.006	0.987–1.024
Marital union	Married (reference value)								
	Unmarried	5.731	0.561–3.338	0.921	0.207–2.206	1.067	0.316–1.961	1.139	0.704–1.844
	Extramarital union	7.038	0.183–2.535	1.037	0.139–4.542	1.448	0.283–4.217	1.122	0.363–3.462
	Divorced	4.200	0.455–3.042	0.747	0.196–1.885	1.559	0.746–4.459	0.321	0.092–1.113
	Widowed	6.857	0.13–1.491			2.027		3.365	0.637–17.793
Number of children	(reference value)								
	1	1.088	0.594–3.399	1.586	0.403–4.463	0.884	0.299–1.775	0.386	0.415–1.314
	2	0.880	0.526–2.847	0.891	0.249–2.462	0.884	0.314–1.834	0.907	0.637–1.682
	3	0.731	0.281–2.978	0.500	0.116–2.108	0.952	0.238–2.770	0.685	0.293–2.099
Education	3 years of secondary school (reference value)								
	4 years of secondary school	0.944	0.582–1.613	0.663	0.323–1.285	1.050	0.631–1.805	0.927	0.635–1.661
	Higher education −2-years of vocational studies	1.612	0.492–5.571	0.682	0.165–2.829	3.698	1.354–12.598	0.813	0.415–3.236
	Higher education-3 years of vocational or academic studies	1.343	0.380–4.740	0.577	0.093–1.740	2.219	0.753–7.699	0.939	0.303–2.966
	University	2.364	0.864–7.937	0.692	0.195–2.067	1.525	0.550–3.539	0.693	0.536–2.765
Managerial position	No (reference value)								
	Yes	0.813	0.397–1.397	1.049	0.434–2.118	1.293	0.356–1.306	0.980	0.546–1.759

OR, Odds ratio; 95% CI, 95% Confidence interval.

Table 2 shows the results of EE, DP, and PA subscales and for the total burnout, modeling with the application of multivariate logistic regression analysis in regard to the socio-descriptive characteristics as independent variables: (sex, age, marital union, number of children, education, and managerial position).

**Table 2:** Average scores of the eight domains of the SF-36 questionnaire.

Domains	*N*	Minimum	Maximum	x̄	SD
PF	353	35.00	100.00	93.68	9.31
RF	353	0.00	100.00	74.50	35.62
RE	353	0.00	100.00	85.08	26.30
VT	353	20.00	100.00	62.15	17.19
MH	353	44.00	100.00	80.00	10.61
SF	353	25.00	100.00	82.11	15.77
BP	353	22.50	100.00	85.13	18.25
GH	353	5.00	100.00	66.60	19.27

PF, Physical functioning; RF, Physical role; RE, Emotional role; VT, Vitality; MH, Mental health; SF, Social functioning; BP, Body pain; GH, General health. $\bar{X}$ , Arithmetic mean; SD, Standard deviation.

The independent variable “sex” was found to be significant at the *p* = 0.10 level; its cross-ratio (i.e., the male category in relation to the female reference category) was 0.277, while its corresponding 95% confidence interval (CI) for the cross-ratio was 0.098–0.780. On the basis, it was determined that employed men were 82% less likely to exhibit EE compared to employed women.

The independent variable “age” of the employees also showed a significant effect on a high degree of EE in the examined group (*p* < 0.001). The chances of an employee having a high degree of EE increased with age, at a ratio of 0.962, (95% CI: 0.946–0.979). With each additional year of life, EE increased by 3.8%. The effect of dependent variables on the independent variable (i.e., level of depersonalization) was not determined.

The reference category was represented by high and moderate levels of PA. The sex of the respondents stood out as a significant variable in the dependent PA variable modeling. The cross-ratio of “male” in comparison to the reference category “female” amounted to 2.644, significant at the level of *p* = 0.10, while its corresponding 95% confidence interval for the cross-ratio was 0.879–7.954. The probability that male responders manifested burnout in the form of lowered PA was reduced by 164% in comparison to female respondents.

The respondents’ education also stood out as a significant variable in the dependent PA variable. The cross-ratio of “university” education in comparison to the reference category of “3 years of secondary school” amounted to 3.698, significant at the level of *p* = 0.10, while its corresponding 95% CI for the cross-ratio amounted to 1.237–11.051. The probability that responders with university education manifested the burnout in the form of lowered PA was reduced by 269% in comparison to the respondents with 3-year of secondary school.

Table 2 shows the average scores of the basic eight domains of the SF-36 questionnaire.

Quality of life is a subjective dimension whose evaluation is extremely important in the identification of risks to employees’.

Physical functioning (PF) was the domain with the highest average score (93.68), while the average scores for vitality (VT): 62.15, and that for general health (GH): 66.60, were the lowest (Table 3).

**Table 3:** Composite PHC score in relation to the socio-descriptive characteristics of employees.

Characteristics		*N*	x̄	SD	SEM	95% CI		Min	Max
						Lower	Upper		
Sex	Male	330	79.35	17.75	0.97	77.43	81.27	27.50	100.00
	Female	23	88.99	11.79	2.45	83.89	94.09	49.38	100.00
	*F* = 6.574; *p* = 0.011								
Marital union	Married	227	76.72	18.34	1.21	74.32	79.12	27.50	100.00
	Unmarried	87	88.23	12.78	1.37	85.50	90.95	38.75	100.00
	Extramarital union	12	91.19	10.64	3.07	84.43	97.95	64.38	100.00
	Divorced	23	79.05	12.65	2.63	73.58	84.52	57.50	100.00
	Widower/widow	4	56.87	24.91	12.4	17.22	96.53	34.38	92.50
	*F* = 10.785; *p* = 0.001								
Number of children	0	120	86.76	13.36	1.22	84.35	89.18	38.75	100.00
	1	84	79.65	17.94	1.95	75.76	83.55	33.75	100.00
	2	129	74.74	18.61	1.63	71.49	77.98	27.50	100.00
	3	20	74.44	18.79	4.20	65.64	83.23	42.50	96.25
	*F* = 11.382; *p* = 0.001								
Education	3 years of high school	103	78.99	17.57	1.73	75.55	82.42	33.75	100.00
	4 years of high school	193	79.80	17.80	1.28	77.27	82.32	33.75	100.00
	Higher education-2 years of vocational studies	16	72.85	19.72	4.93	62.34	83.36	27.50	97.50
	Higher education-3 years of vocational or academic studies	14	84.15	18.12	4.84	73.68	94.61	48.75	100.00
	*F* = 2.076; *p* = 0.083								
Work in shifts Work hours Workplace	Yes	288	78.72	18.26	1.07	76.60	80.84	27.50	100.00
	No	65	85.54	12.82	1.59	82.37	88.72	55.00	100.00
	*F* = 8.157; *p* = 0.005								
	≤8 h	36	84.96	13.39	2.23	80.43	89.50	55.63	97.50
	8–12 h	277	79.71	17.68	1.06	77.61	81.80	27.50	100.00
	≥12 h	40	77.37	19.56	3.09	71.11	83.63	33.75	100.00
	*F* = 1.931; *p* = 0.147								
	Boss of security service	53	85.81	12.79	1.75	82.28	89.34	54.38	100.00
	Security officer	300	78.95	18.11	1.02	76.89	81.00	27.50	100.00
	*F* = 6.986; *p* = 0.009								

$\bar{X}$ , Arithmetic mean; SD, Standard deviation; SEM, Standard error of mean; 95% CI, 95% Confidence interval; Min, Minimum value; Max, Maximum value; and F, ANOVA.

Table 3 shows a composite score of PHC in relation to the socio-descriptive characteristics of employees.

Compared to men, a significantly higher PHC score was observed in women (*p* = 0.011), as well as in unmarried participants (*p* = 0.001). PHC score was significantly higher in employees without children (*p* = 0.001), compared to employees with children. Employees working in shifts had significantly higher average values of PHC score (*p* = 0.005). Working hours had no significant impact on the PHC score (*p* = 0.147), whereas the managerial position had a significant impact on increasing PHC score (*p* = 0.009).

Table 4 shows the composite MHC score in relation to the socio-descriptive characteristics of employees.

**Table 4:** Composite MHC score in relation to socio-descriptive characteristics of employees.

Characteristics		*N*	x̄	SD	SEM	95% CI		Min	Max
						Lower	Upper		
Sex	Male	330	77.00	13.00	0.71	75.59	78.41	30.38	100.00
	Female	23	82.11	14.41	3.00	75.88	88.35	48.71	100.00
	*F* = 3.275; *p* = 0.071								
Marital union	Married	227	76.53	12.22	0.81	74.93	78.13	30.38	100.00
	Unmarried	87	80.99	13.77	1.47	78.05	83.93	33.75	100.00
	Extramarital union	12	76.85	18.13	5.23	65.32	88.37	37.00	95.25
	Divorced	23	75.42	12.78	2.66	69.89	80.95	39.46	95.50
	Widower/widow	4	55.63	8.10	4.05	42.73	68.53	48.71	66.50
	*F* = 4.961; *p* = 0.001								
Number of children	0	120	79.55	13.49	1.23	77.11	81.99	33.75	100.00
	1	84	78.06	13.99	1.52	75.03	81.10	39.46	100.00
	2	129	75.17	12.48	1.09	72.99	77.34	30.38	100.00
	3	20	74.93	9.06	2.02	70.69	79.17	56.33	91.00
	*F* = 2.653; *p* = 0.049								
Education	3 years of high school	103	103	77.18	12.8	1.26	74.68	79.690	40.13
	4 years of high school	193	193	76.99	13.4	0.96	75.09	78.903	30.38
	Higher education-2 years of vocational studies	16	16	74.96	14.7	3.69	67.07	82.850	37.00
	Higher education-3 years of vocational or academic studies	14	14	81.19	13.7	3.67	73.26	89.129	48.42
	*F* = 0.692; *p* = 0.598								
Work in shifts	Yes	288	76.73	13.45	0.79	75.17	78.29	30.38	100.00
	No	65	80.00	11.36	1.40	77.19	82.82	53.75	100.00
	*F* = 3.305; *p* = 0.070								
Work hours	≤8 h	36	79.14	11.14	1.85	75.37	82.91	53.75	96.50
	8–12 h	277	77.60	12.83	0.77	76.08	79.11	30.38	100.00
	≥12 h	40	73.89	16.29	2.57	68.68	79.11	39.46	96.75
	*F* = 1.773; *p* = 0.171								
Workplace	Boss of security service	53	78.88	12.25	1.68	75.50	82.25	50.88	100.00
	Security officer	300	77.06	13.29	0.76	75.55	78.57	30.38	100.00
	*F* = 0.857; *p* = 0.355								

$\bar{X}$ , Arithmetic mean; SD, Standard deviation; SEM, Standard error of mean; 95% CI, 95% Confidence interval; Min, Minimum value; Max, Maximum value; and F, ANOVA.

There was no significant difference in the average MHC score in relation to the sex of employees (*p* = 0.071). Widows/widowers had a significantly lower MHC score compared to employees with different marital status (*p* = 0.001). Employees without children or with one child had a significantly higher MHC score compared to employees with two or three children (*p* = 0.049). The level of education had no significant impact on the average MHC score (*p* = 0.598). Work in shifts (*p* = 0.070), working hours (*p* = 0.171), and managerial position (*p* = 0.355) had no significant impact on the average MHC score.

The mean total quality of life (TQL) score was 78.66 ± 13.77. The mean composite score was 79.98 ± 17.57 for PHC and 77.33 ± 13.14 for MHC.

Table 5 shows the TQL score in relation to the socio-descriptive characteristics of employees.

**Table 5:** TQL score in relation to the socio-descriptive characteristics of employees.

Characteristics		*N*	x̄	SD	SEM	95% CI		Min	Max
						Lower	Upper		
	Female	23	85.55	11.90	2.48	80.41	90.70	49.04	100.00
	*F* = 6.260; *p* = 0.013								
Age	Male	330	44.09	11.44					
	Female	23	36.91	7.92					
	*F* = 8.752; *p* = 0.003								
	Married	227	76.63	13.64	0.90	74.84	78.41	33.63	100.00
	Unmarried	87	84.61	12.25	1.31	82.00	87.22	36.25	100.00
	Extramarital union	12	84.02	13.01	3.75	75.75	92.29	56.00	97.00
	Divorced	23	77.23	11.48	2.39	72.26	82.20	54.42	95.25
	Widower/widow	4	56.25	14.10	7.05	33.80	78.70	42.31	74.79
	*F* = 9.248; *p* = 0.001								
Number of children	0	120	83.16	11.94	1.09	81.00	85.32	36.25	100.00
	1	84	78.86	14.67	1.60	75.67	82.04	36.69	100.00
	2	129	74.95	13.93	1.22	72.52	77.38	33.63	100.00
	3	20	74.68	11.61	2.59	69.25	80.12	52.02	93.63
Education	3 years of high school	103	78.09	13.77	1.35	75.39	80.78	36.94	100.00
	4 years of high school	193	78.39	13.94	1.00	76.41	80.37	33.63	100.00
	Higher education-2 years of vocational studies	16	73.90	15.14	3.78	65.84	81.97	41.71	97.13
	Higher education-3 years of vocational or academic studies	14	82.67	15.52	4.14	73.71	91.63	48.58	97.75
Work in shifts	Yes	288	77.73	14.32	0.84	76.06	79.39	33.63	100.00
	No	65	82.77	10.11	1.25	80.27	85.28	56.46	100.00
	*F* = 7.247; *p* = 0.007								
Work hours	≤8 h	36	10.20	10.06	1.67	78.64	85.46	64.38	97.00
	8–12 h	277	78.47	13.75	0.82	77.02	80.28	33.63	100.00
	≥12 h	40	11.33	16.18	2.55	70.46	80.81	36.69	97.63
	F = 2.069; *p* = 0.128								
Workplace	Boss of security service	53	12.00	10.09	1.38	79.56	85.12	57.90	100.00
	Security officer	300	78.00	14.24	0.82	76.39	79.62	33.63	100.00
	*F* = 4.513; *p* = 0.034								

$\bar{X}$ , Arithmetic mean; SD, Standard deviation; SEM, Standard error of mean; 95% CI, 95% Confidence interval; Min, Minimum value; Max, Maximum value; and F, ANOVA.

*Compared to women*, men had a significantly lower average score of total quality of life (*p* = 0.013). Married employees had a significantly lower TQL score compared to those who were not married (*p* = 0.001). Widowed employees had the statistically lowest TQL score. Employees without children or with one child had a significantly higher TQL score, compared to employees with two, three, or more children (*p* = 0.001).

Education did not have a significant impact on the average TQL score (*p* = 0.160). Shift work has a significant impact on the reduction in average TQL score (*p* = 0.007). Employees in managerial positions had a significantly higher TQL score compared to those who did not hold managerial positions (*p* = 0.034).

The first hypothesis proposed that males are at higher risk of develop burnout than females but males had higher PA subscale. Females were at higher risk for total burnout and EE than males (*p* < 0.001). Males had significantly decreased quality of life.

The second hypothesis indicated that older age is in the significant relationship with the higher subscale EE of the burnout (*p* < 0.001).

Hypothesis 3 indicated that employees who are married show lower levels of the quality of life than unmarried employees. ANOVA (*F* = 9.248; *p* = 0.001) showed that employees who are married have significantly decreased quality of life, and the further *post hoc* analysis confirmed the same.

The fourth hypothesis proposed that employees without children or with one child had a significantly higher MHC score and total quality of life, compared to employees with two, three, or more children. Employees without children or with one child had a significantly higher MHC score compared to employees with two or three children (*p* = 0.049).

The fifth hypothesis proposed significant positive relationship between managerial position and higher level of education with higher PA subscale and a lower risk of total burnout the quality of life. The managerial position had a significant impact on increasing PHC score (*p* = 0.009) and on the total quality of life compared to employees who did not hold managerial positions (*p* = 0.034).

The sixth hypothesis proposed that shift work has a significant impact on the reduction in total quality of life (*p* = 0.007). Long working hours are in the significant negative relationship with the decreased quality of life. Working hours from 8 to 12 h per day based on ANOVA (*F* = 2.069; *p* = 0.128) are significantly decrease the quality of life (Table 6).

**Table 6:** Spearman correlation of the total burnout and the subscales of the burnout EE, DP, PA with the PHC and MHC composite scores, and total QOL of the SF-36 questionnaire.

Statistical values			PHC	MHC	TQL
Total burnout	*r*		−0.265^**^	−0.391^**^	−0.351^**^
	*p*		0.000	0.000	0.000
	*N*		353	353	353
	Bootstrap^c^	Bias	0.000	0.000	0.000
		SE	0.050	0.046	0.048
		95% CI	−0.361	−0.480	−0.445
			−0.163	−0.301	−0.258
Emotional exhaustion (ЕЕ)	*r*		−0.311^**^	−0.434^**^	−0.393^**^
	*p*		0.000	0.000	0.000
	*N*		353	353	353
	Bootstrap^c^	Bias	−0.001	0.000	−0.001
		SE	0.051	0.043	0.047
		95% CI	−0.407	−0.516	−0.482
			−0.205	−0.344	−0.297
Depersonalization (DP)	*r*		−0.231^**^	−0.331^**^	−0.302^**^
	*p*		0.000	0.000	0.000
	*N*		353	353	353
	Bootstrap^c^	Bias	−0.002	−0.002	−0.002
		SE	0.049	0.049	0.048
		95% CI	−0.327	−0.433	−0.402
			−0.140	−0.233	−0.212
Personal accomplishment (PA)	*r*		0.145^**^	0.230^**^	0.200^**^
	*p*		0.006	0.000	0.000
	*N*		353	353	353
	Bootstrap^c^	Bias	−0.001	−0.001	−0.001
		SE	0.055	0.052	0.054
		95% CI	0.033	0.123	0.094
			0.253	0.328	0.305

**Statistically significant; *r*_s_, Spearman correlation coefficient; SE, Standard error; 95%CI, 95% confident interval; Bootstrap, Internal validation.

*Hypothesis 7*: There is a significant negative correlation between job burnout and the quality of life of the security employees.

A significant negative correlation was found between total burnout and PHC with Spearman correlation coefficient (*r*_s_) of −0.265 (95%CI from −0.361 to −0.163), between total burnout and MHC with *r*_s_, of −0.391 (95%CI from −0.480 to −0.301) and between total burn and TQL with *r*_s_, of −0.351 (95%CI from −0.445 to −0.258).

There is a significant negative correlation between EE and PHC with *r*, of −0.311 (95%CI from −0.407 to −0.205), between EE and MHC with *r*, of −0.434 (95%CI from −0.516 to −0.344) and between EE and TQL with *r*, of −0.393 (95%CI from −0.482 to −0.29 o 7).

A significant negative correlation between DP and PHC with *r*, −0.231 (95%CI from −0.327 to −0.140), between DP and MHC with *r*, of −0.331 (95%CI from −0.433 to −0.233), and between DP and TQL with *r*, of −0.302 (95%CI from −0.402 to −0.212).

There is a significant positive correlation between PA and PHC with *r*, of 0.145 (95%CI from 0.033 to 0.253), between PA and MHC with *r*, of 0.230 (95 from %CI 0.123 to 0.328), and between PA and TQL with *r*, of 0.200 (95% CI from 0.094 to 0.305).

## 4 Discussion

Private security refers to the security protection services that private security organizations provide to control crimes, protect lives and other assets, and maintain order at their employers’ facilities (4–6). Working in the private security sector is most similar to police work in most countries (5, 11) and the basic difference between public police officers and private security officers is reflected in the fact that public police officers have legal power delegated by the state. On the other hand, private security employees do not have the status of authorized officers.

Security work is considered a male occupation in many countries, and this is no different in Serbia; thus, the sex differences were expected. Our study show that female sex and older age were associated with a higher risk of total burnout and the development of EE subscale. The first hypothesis is partially confirmed. The second hypothesis is confirmed. We found a significant association of socio-descriptive characteristics such as male sex, higher education, and managerial position with higher PA subscale and a lower risk of total burnout. We did not found significant association of socio-descriptive characteristics and DP subscale.

According to presented results, male sex, marital union, two or more children, and direct contact with clients were significantly associated with a lower total quality of life of the employees. Hypotheses 1, 3, 4, and 5 are confirmed. Shift work significantly reduced the total quality of life, while managerial positions increased the quality of life of the examined employees. We confirmed hypotheses 5 and 6.

About one-third of responders had symptoms of total burnout syndrome. A much higher number of employees developed a moderate or high level of burnout symptoms on individual subscales: EE (high 66.3%, moderate 19.8%, and low 13.9%); DP (high 82.4%, moderate 16.2%, and low 1.4%); and PA (low 34.5%, moderate 32.9%, and high 36.2%). All values of total burnout and EE and DP subscales were higher in our examined population than in Brazilian similar population, only was lower PA subscale in our responders: EE 46, 25, and 29%; DP 21, 30, and 49%; PA 41, 31, and 28% (45), and in Mexican too, a prevalence of total burnout was 23.36% of the Mexican police officers, high EE in 44.16%, DP in 49.29%, and PA in 41.03% (20).

Our findings are compatible with published studies that demonstrate an increased risk of burnout among women (23–25, 36, 46). In our study, the male sex of the employees also stood out as a significant variable for the higher PA subscale. In the study of Adecola et al. (47) males had higher level of PA subscale than women although this subscale was reduced in both sexes. There have also been different results in the literature according to which men are protected from EE if they are in an emotional relationship, while women are at risk of developing EE if they have a family (19, 35, 36). In one study, similar to ours, the EE subscale of burnout presented the highest score in participants (47).

State police officers who were in an emotional relationship were more prone to EE but experienced higher PA, and there was no significant difference between state police officers in the civil service who had children or not, with regard to the development of burnout (16, 21, 30, 35, 36). Employed women, due to their responsibilities toward their family and work, have difficulties in harmonizing professional and private obligations. In certain studies, women were found to be more prone to EE (33, 36), while men were more susceptible to DP.

Our findings are compatible with published studies that demonstrated an increased risk of job burnout with increased age. Results of a study performed on the professional military personnel of the Serbian Armed Forces (46) showed that the highest level of burnout was measured on the subscales EE in military personnel aged 23–30 years of age (*p* < 0.05). De Solana reported that being younger contributed to increased burnout levels (18).

In relation to marital status, employees who were unmarried seemed to be more exposed to burnout compared to those who lived with a partner, in our study. However, such findings seem to be more appropriate in men, as, in the case of working women, it constitutes an additional risk factor, since working women are usually responsible for household chores, therefore, this may pose a difficulty in reconciling personal and professional life (21, 22, 34, 36). No significant association with burnout was found (10). This finding is linked to the greater emotional support supplied by the partner or his/her children. Married employees can also share living expenses with a partner and in this way lessen the feeling of financial responsibility.

The number of children in a family can also play an important role in appearance of burnout. In a study by Brady et al. (33), a higher number of children was correlated with a lower level of burnout. According to the findings of Dutch researchers that the presence of young children and doing more household chores were positively related to feelings of burnout, whereas having children reduced employees’ feelings of burnout (32). Having children is associated with lower PA (16, 29, 32, 36). Our study showed that employees without children or with one child had a significantly higher MHC score and total quality of life, compared to employees with two, three, or more children. Based on our results, a managerial position and higher education were protective factors in relation to the development of burnout. The hypothesis 5 is confirmed. In our study, ≤10% of participants had a university degree. In a similar study, conducted with state police officers in Spain, there were almost twice as many employees with a university degree, compared to employees from our study but no significant relationships were found between educational level and burnout in this study (29). According to our results, risk of appearance of job burnout decreased with the increase in academic qualifications, which is consistent with the results from the literature (14, 28, 31, 48). In EU countries, there are significantly more employees in private security sector with higher education and university degrees (12, 13, 33, 49–51) than in Serbia.

According to the findings of Ronen and Pines (19), te Brake et al. (21), and Maccaaro (22), male sex, shift work, and a low score in relationships with superiors at the work-place were connected with a higher rate of burnout than females. According to results of similar studies, women have a greater tendency to utilize emotion focused coping their manner of support, leading to greater work–family conflict (36, 52).

A possible explanation could be the fact that managers are less exposed to direct contact with clients and potential attackers; moreover, they do not usually work in shifts and have shorter working hours. They also don’t have an obligation to carry a weapon at the workplace. Educated individuals with more work experience have more self confidence in the performance of their duties; they show greater self-control in contact with various stressors and experience less emotional trauma (16, 18, 22, 53).

Job burnout affects the quality of life (QOL) of employees and some socio-descriptive characteristics, too. According to our findings, male sex, marital union, two or more children, and direct contact with clients were significantly associated with a lower quality of life in security employees. The hypothesis 7 is confirmed. Despite married employees having a low total quality of life, in our study, widowed employees had the lowest quality of life. While shift work significantly decreased the total quality of life, managerial positions increased the total quality of life of the employees. We found a significant negative correlation between job burnout and the quality of life of the security employees. Only PA subscale was inversely correlated with the quality of life. Correctional officers in Brazil showed the presence of both burnout and reduced quality of life (45). De Moraes et al. (54) presented that 19.2% of their total sample had high levels of burnout, and the same participants also exhibited reduced quality of life (54).

There are inconsistent findings on police officers’ sex and perceived quality of life. A Greek study suggests that female and male police officer’s scores do not differ significantly overall, but females reported lower levels of subscales of health-related quality of life which is—like job satisfaction—negatively associated with perceived stress. However, significant interactions were not revealed (55). Other studies have found no significant sex differences concerning police officers’ quality of life (48–51, 56, 57). However, research on police officers’ quality of life is only recently emerging (50–52, 58). The emphasis on job burnout prevention by the state authorities and workers in the EU shows that burnout syndrome is a problem that confers an economic burden (53); in the context of employee’s effectivity and health, it has to be dealt with. With such a perspective, the European Agency for Safety and Health at Work (EU-OSHA) supports the prevention of occupational stress-related disorders thought the campaign: “Healthy workplaces manage stress” (53).

The possibility of developing occupational burnout among employees in the professional private security sector in the Central Serbia can be seen in a broader context by examining general values, political and economic opportunities, and instability in the region. Bearing in mind the specifics of the job, an examination of the socio-descriptive characteristics of candidates before employment is necessary, which would enable the identification of persons at higher risk for burnout. In this way, a better selection of candidates for security jobs can be established, and the risk of burnout can be prevented or reduced, while mitigating the negative effects on employees quality of life. Monitoring employee job satisfaction, continuously improving work organization, continuously educating-employees in security work, and bringing awareness to burnout symptoms can enable timely treatment, rehabilitation, temporary job changes, or, permanent job changes, with the aim of preserving the health and life of employees.

As a strength, this is the first study of burnout and the quality of life in professional private security employees in Central Serbia. The employees were interested in this topic and willingly participated in the study. According to the results obtained in our study, the following factors bother employees the most: working in shifts and working 8–12 h a day.

There were several limitations of our study. Only cross-sectional data were used; hence, no causal relationships could be identified. The quality of life was self-assessed, which may have led to desirability bias. The possible influence of significantly higher number of males than females in the study.

Practical applications of the findings of our study in the daily work of security employees can be implemented immediately after its completion. All employees with a high score of total burnout can be referred to a psychologist and, if necessary, to a psychiatrist. An occupational medicine specialist can be included in the consultation with employees. Employees may change workplace, whether temporary, but if necessary, or permanent, according to a psychologist’s advice. Security officers whose biggest problem is working in shifts can be transferred to the workplace only for a daily shift and working hours can also be reduce in some employees. In many cases, private security officers receive inadequate training (18).

## 5 Conclusion

On the basis of the presented results, female sex and older age were associated with higher scores of occupational burnout and higher levels emotional exhaustion. Male sex, higher education, and managerial position were associated with higher personal achievement and a lower risk of total burnout. Socio-descriptive characteristics such as male sex, marital union, two or more children, and direct contact with clients were significantly associated with a lower quality of life of the employees. Shift work significantly reduced the total quality of life, while managerial positions increased the quality of life of the examined employees. Occupational burnout negatively correlated with the quality of life of employees. According to our results, employees with a higher score of total burnout or a particular (emotional exhaustion or depersonalization) subscale were partaking in psychological consultation, with a suggestion to temporarily or permanently change the workplace. The accelerated development of the professional private security sector in Central Serbia requires additional research on burnout and quality of life in this group of employees.

## Data availability statement

The original contributions presented in the study are included in the article/supplementary material, further inquiries can be directed to the corresponding author/s.

## Ethics statement

The studies involving humans were approved by the Ethics Committee of the Faculty of Medicine of the University of Pristina with temporary seat in Kosovska Mitrovica by the Decision No 09-972-1 dated September 10, 2018. The studies were conducted in accordance with the local legislation and institutional requirements. The participants provided their written informed consent to participate in this study.

## Author contributions

NR: Conceptualization, Investigation, Writing – original draft. DV: Conceptualization, Data curation, Investigation, Methodology, Writing – review & editing. MM: Conceptualization, Methodology, Supervision, Writing – review & editing. LK: Conceptualization, Methodology, Supervision, Writing – review & editing. VJ: Methodology, Supervision, Writing – review & editing. BS: Data curation, Investigation, Methodology, Writing – review & editing. NM: Formal Analysis, Methodology, Writing – review & editing. VC: Data curation, Investigation, Validation, Writing – review & editing. EM-Z: Data curation, Investigation, Project administration, Writing – review & editing. SG: Data curation, Formal Analysis, Investigation, Writing – review & editing. ĐG: Data curation, Investigation, Formal Analysis, Writing – review & editing. OĐ: Data curation, Investigation, Software, Writing – review & editing. MS: Data curation, Investigation, Resources, Writing – review & editing. NB: Data curation, Investigation, Project administration. DM: Data curation, Investigation, Writing – review & editing. SO: Conceptualization, Supervision, Writing – review & editing.
